# Obituary

**Published:** 1859-06

**Authors:** 


### OBITUARY.

DIED—At the residence of James Lewis, near Georgetown, Pettis county, Missouri, on the 29th April, Dr. OSCAR FORGERSON, aged about 26 years.

The numerous friends of Dr. Forgerson will regret to learn that he died four days after arriving at his destination in Missouri.

He was, for several years, a citizen of Peru, engaged in the practice of his profession. He gained, and retained, the confidence of all who knew him.

With the notice of his death, comes the saddening remembrance of his suffering, which he bore with a fortitude seldom equalled.

In the prime of early manhood; thorough in the knowledge of his profession; he came among us with the buoyant hope of a long, useful and happy life. Stricken by that insidious destroyer of human life—consumption—his fortitude and manliness availed but to prolong, not to relieve his suffering.

In his death there is a lesson for the living. Vigorous and joyous to-day, to-morrow stricken by that remorseless tyrant, death: the third day, we are numbered among those who have been, but are not. Let us ponder upon, and improve the lesson.

To us, there was something touching in the devotion he manifested toward the companion of his life's voyage, to whom he had been married but a short time. She too, was sinking under the influence of the same disease, the result, in part, of her attention to, and her sympathy with him. His last and only earthly desire was to place her under the kind and tender care of her mother. Circumstances favored this desire, and they started for the interior of Missouri, where her parents lived. Arriving, the mother met him. In an inarticulate voice he whispered, "Mother, the girl you gave me, I return to you. While I was well, I cared for her. Now, I bring her back, and am come myself to die!"

True to the pledge he made, when he could no longer be her protector, he gave her back and died.

This, his last desire, was gratified: and he now sleeps the sleep that knows no waking, beneath the prairie turf.

